# Comparison of early warning scores for sepsis early identification and prediction in the general ward setting

**DOI:** 10.1093/jamiaopen/ooab062

**Published:** 2021-08-02

**Authors:** Sean C Yu, Nirmala Shivakumar, Kevin Betthauser, Aditi Gupta, Albert M Lai, Marin H Kollef, Philip R O Payne, Andrew P Michelson

**Affiliations:** 1 Institute for Informatics, Department of Medicine, Washington University School of Medicine in St. Louis, St. Louis, Missouri, USA; 2 Department of Biomedical Engineering, Washington University School in St. Louis, St. Louis, Missouri, USA; 3 Department of Medicine, Washington University School of Medicine in St. Louis, St. Louis, Missouri, USA; 4 Department of Pharmacy, Barnes-Jewish Hospital, St. Louis, Missouri, USA; 5 Division of Pulmonary and Critical Care, Department of Medicine, Washington University School of Medicine in St. Louis, St. Louis, MO, USA

**Keywords:** sepsis, early warning score, predictive analytics

## Abstract

The objective of this study was to directly compare the ability of commonly used early warning scores (EWS) for early identification and prediction of sepsis in the general ward setting. For general ward patients at a large, academic medical center between early-2012 and mid-2018, common EWS and patient acuity scoring systems were calculated from electronic health records (EHR) data for patients that both met and did not meet Sepsis-3 criteria. For identification of sepsis at index time, National Early Warning Score 2 (NEWS 2) had the highest performance (area under the receiver operating characteristic curve: 0.803 [95% confidence interval [CI]: 0.795–0.811], area under the precision recall curves: 0.130 [95% CI: 0.121–0.140]) followed NEWS, Modified Early Warning Score, and quick Sequential Organ Failure Assessment (qSOFA). Using validated thresholds, NEWS 2 also had the highest recall (0.758 [95% CI: 0.736–0.778]) but qSOFA had the highest specificity (0.950 [95% CI: 0.948–0.952]), positive predictive value (0.184 [95% CI: 0.169–0.198]), and F1 score (0.236 [95% CI: 0.220–0.253]). While NEWS 2 outperformed all other compared EWS and patient acuity scores, due to the low prevalence of sepsis, all scoring systems were prone to false positives (low positive predictive value without drastic sacrifices in sensitivity), thus leaving room for more computationally advanced approaches.

## Lay Summary

Sepsis is a syndrome caused by an infection resulting in organ dysfunction and high rates of death, is implicated in nearly half of all inpatient deaths, and is the costliest inpatient condition in the United States. Early recognition and treatment are critical to the management of septic patients. As a result, over time, researchers have developed numerous early warning scores that use clinical measurements such as vital signs and lab results to generate a value that is indicative of the severity of illness and is predictive of clinical deterioration. Increasingly, these scores have been used as screening tools for sepsis management. To understand the comparative performance of these early warning scores in the general ward setting, electronic health records data were used to calculate the scores. Of the compared scores, the National Early Warning Score (NEWS 2) outperformed the rest. However, partially due to the low prevalence of sepsis in the general ward, even NEWS 2 was prone to false positives, highlighting the potential for improvement using more advanced computational methods.

## BACKGROUND AND SIGNIFICANCE

Sepsis is the dysregulated host response to infection that can lead to life-threatening organ failure.^1^ It is a deadly disease process that contributes to nearly 50% of all inpatient deaths and is the most expensive inpatient condition paid for by the US healthcare system, totaling $24 billion on an annual basis.^2^^,^^3^ Early recognition and effective antimicrobial therapy are the cornerstones of sepsis management, but timely detection remains a clinical challenge.^4^^,^^5^

Several approaches to early sepsis identification have been linked to key physiologic derangements commonly seen during disease progression. The previously used Systemic Inflammatory Response Syndrome (SIRS) criteria which graded the host’s response to an inflammatory insult were easy to use at the bedside, but nearly half of all inpatients met these criteria during their hospitalization.^6^ As a result, the SIRS criteria have been criticized for being overly sensitive, which greatly limited its utility as a sepsis surveillance tool.^6^ The most recent sepsis consensus statement introduced the quick Sequential Organ Failure Assessment (qSOFA) as a mortality risk stratification tool, but qSOFA was not validated as a sepsis surveillance tool.^1^^,^^7^

One emerging approach to sepsis screening is to implement early warning scores (EWS), such as the Modified Early Warning Score (MEWS), the National Early Warning Score (NEWS), or its successor, the NEWS 2.^5^ These scores grade the severity of physiologic derangement and provide a well-validated means of assessing risk for all-cause clinical deterioration. Other patient acuity scoring systems, also based on physiological measurements, such as Acute Physiology and Chronic Health Evaluation (APACHE II) have been used longitudinally for risk stratification.^8^ Although many hospital systems are starting to deploy these EWS to aid in sepsis screening on the general ward, they have not been validated or directly compared for this purpose and their performances remain unknown.^5^^,^^9–11^ The objective of this study was to evaluate and compare the performance of commonly used EWS on sepsis surveillance for patients admitted to the general ward.

## MATERIALS AND METHODS

### Study design, data sources, and population

All patients ≥18 years of age admitted to Washington University in St. Louis/Barnes-Jewish Hospital between January 1, 2012 and June 1, 2018 were eligible for inclusion. Patients were excluded if discharged < 12 h after sepsis onset, total length of stay was < 48 h, surgery was performed in the preceding 72 h, < 1 set of vital signs were recorded in the 24-h preceding index time, or if < 1 set of common labs results (creatinine and white blood cell count) were recorded in the 24-h preceding index time. Patients were excluded if sepsis was present on admission or if admission service was hospice, psychiatry, or obstetrics and gynecology due to the highly variable rates of physiologic data collection. Patients were also excluded if they no encounter billing code, vital sign, laboratory, service, room, or medication data to indicate a complete hospitalization. To ensure temporal similitude between cohorts, patient encounters <12 h or >14 days in duration were excluded. Electronic health record (EHR) data were extracted from the Research Data Core at Washington University in St. Louis School of Medicine. This project was approved with a waiver of informed consent by the Washington University in St. Louis Institutional Review Board (IRB#201804121).

### Sepsis criteria

Sepsis was defined according to the Sepsis-3 consensus statement as suspicion of infection (SOI; culture collection followed by antibiotics within 72 h or antibiotics followed by culture procurement within 24 h, Supplementary Appendix I) accompanied by a qSOFA score ≥2.^12^ Only the first sepsis event for each patient was evaluated. Time of onset was set as the time of SOI.

### Index time for the nonsepsis cohort

Unlike the sepsis cohort where a specific event—sepsis onset—can be used as the index event, there is no such event for nonsepsis patients. To minimize bias introduced by difference in time-to-index time, nonsepsis patients were subsampled at a ratio of 30:1 and assigned an index-time such that the resultant histograms of time-to-index time (3-h bins) were equivalent (Supplementary Appendix II).

### Early warning scores

The SIRS, MEWS, NEWS, NEWS 2, qSOFA, Sequential Organ Failure Assessment (SOFA), and Acute Physiology And Chronic Health Evaluation (APACHE II) scores were calculated every hour from 12-h prior to index time to 12 h after index time.^7^^,^^13–16^ Scores were calculated using the most abnormal physiological measurement (contributing the most points to the scoring system) as well as the most recent measurement in the 24 h preceding time of measurement. If no values were present in the lookback period, missing values were assumed normal. Additional details on EWS calculations can be found in Supplementary Appendix III. Sensitivity analysis was performed using a lookback period of 12 h. Further, EWS were compared at index time using thresholds defined in previous validation studies on the ability to discriminate between sepsis and non-sepsis patients.^1^^,^^7^^,^^11^^,^^14^^,^^17^ Lastly, EWS were evaluated on their capability for early identification of secondary outcomes: in-hospital mortality within 48 h of index time and the composite outcome of in-hospital mortality or intensive care unit (ICU) transfer within 48 h of index time.

### Statistical analysis

Patient characteristics and outcomes were compared between the sepsis and nonsepsis cohorts using the two-sided Mann-Whitney *U* test or χ^2^ test for numeric and categorical variables, respectively, where *P* < .01 was considered significant. Performance metrics such as the area under the receiver operating characteristic curve (AUROC) and area under the precision recall curves (AUPRC) were reported as the median and 95% confidence interval determined through 1000 sample bootstrap.

## RESULTS

### Population characteristics

In total, 45 776 patients met inclusion criteria and 1496 (3.3%) met sepsis criteria (Table 1 and Supplementary Appendix II). Compared to the nonsepsis population, sepsis patients were slightly older (median [IQR]; 64.3 years [53.4–74.7] vs 60.0 [48.3—70.8], *P* < .01) and more likely to be white (66.6% vs 62.3%, *P* < .01). Sepsis patients also had significantly higher Elixhauser comorbidity scores (16 [8–26] vs 9 [0–17]; *P* < .01), APACHE II scores at the time of sepsis onset (median [IQR]; 13 [10–16] vs 11 [7–14], *P* < .01), longer lengths of stay (median [IQR]; 7.8 [5.3–10.3] vs 4.2 [2.8–6.9]; *P* < .01), and higher rates of in-hospital mortality (12.2% vs 1.1%; *P* < .01).

**Table 1.** Cohort characteristics and outcomes

Variable	Total	Sepsis	Nonsepsis	***P*** ^a^
Number of samples, *n* (%)	45 776 (100.0%)	1,496 (3.3%)	44,280 (96.7%)	**<.01**
Age, median (IQR)	60.2 (48.5–71.0)	64.3 (53.4–74.7)	60.0 (48.3–70.8)	**<.01**
Sex (female), *n* (%)	21,891 (47.8%)	743 (49.7%)	21 148 (47.8%)	.154
Race, *n* (%)	–	–	–	**<.01**
White	28,563 (62.4%)	997 (66.6%)	27,566 (62.3%)	**<.01**
Black	14,303 (31.2%)	378 (25.3%)	13,925 (31.4%)	**<.01**
Asian	323 (0.7%)	9 (0.6%)	314 (0.7%)	.74
Other	2,587 (5.7%)	112 (7.5%)	2,475 (5.6%)	**<.01**
BMI, median (IQR)	27.4 (23.2–32.8)	26.6 (22.5–32.7)	27.4 (23.2–32.8)	**<.01**
Elixhauser comorbidity score, median (IQR)^b^	9 (1–18)	16 (8–26)	9 (0–17)	**< .01**
Time to index time (hours), median (IQR)	47.9 (22.6–94.1)	48.4 (22.4–96.5)	47.9 (22.6–94.0)	.429
APACHE II score at index time, median (IQR)	11 (8–14)	13 (10–16)	11 (7–14)	**<.01**
LOS (days), median (IQR)	4.3 (2.9–7.0)	7.8 (5.3–10.3)	4.2 (2.8–6.9)	**<.01**
Sepsis discharge diagnosis ICD code, *n* (%)	2,236 (4.9%)	243 (16.2%)	1,993 (4.5%)	**<.01**
Discharge disposition, *n* (%)	–	–	–	**<.01**
In-hospital death	658 (1.4%)	182 (12.2%)	476 (1.1%)	**<.01**
Discharge to hospice facility	578 (1.3%)	57 (3.8%)	521 (1.2%)	**<.01**
Discharge to acute care hospital	303 (0.7%)	7 (0.5%)	296 (0.7%)	.436
Discharge to nonacute care facility	6293 (13.7%)	345 (23.1%)	5948 (13.4%)	**<.01**
Discharge to home	37 826 (82.6%)	902 (60.3%)	36 924 (83.4%)	**<.01**
Miscellaneous/other	118 (0.3%)	3 (0.2%)	115 (0.3%)	.853

BMI: body mass index; LOS: length of stay; APACHE: acute physiology and chronic health evaluation; ICU: intensive care unit; ICD: International Classification of Diseases. Bolded values indicate statistical significance using *P* < .01.

^a^ *P*-values were calculated using Mann–Whitney *U* test for continuous and χ^2^ for categorical variables.

^b^ Comorbidity score was calculated using ICD diagnosis codes based on Moore, Med Care, 2017.

### EWS performance

**Figure 1.**
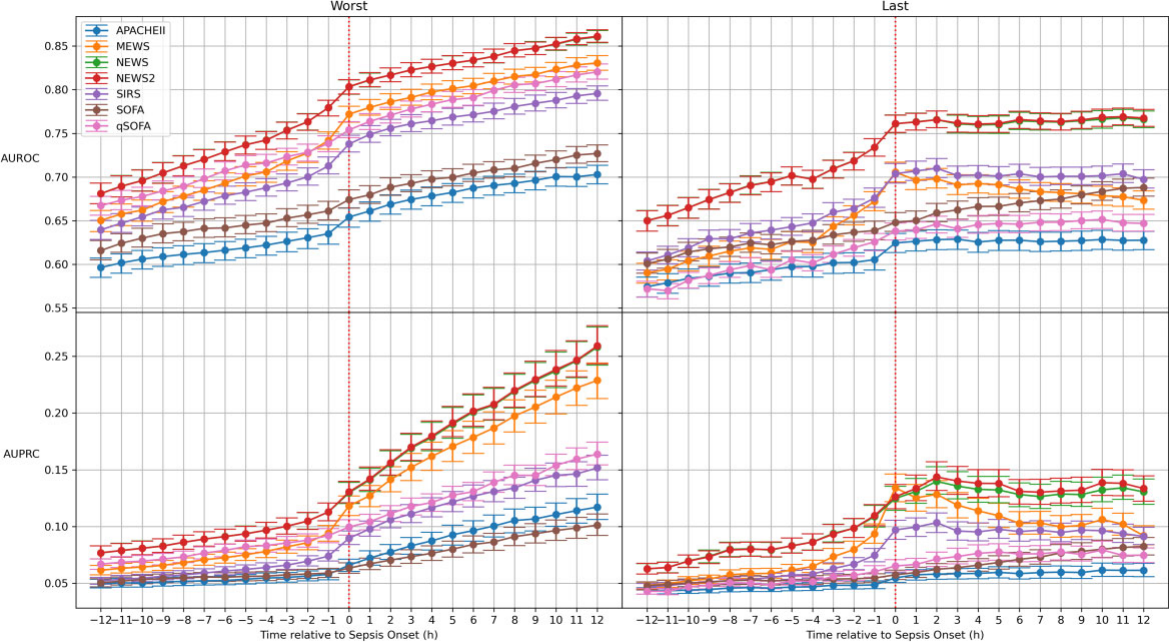
Early warning score performance for sepsis discrimination. SIRS: Systemic Inflammatory Response Syndrome; qSOFA: quick Sequential Organ Failure Assessment; NEWS: National Early Warning Score; MEWS: Modified Early Warning Score; SOFA: Sequential Organ Failure Assessment; APACHE: Acute Physiology and Chronic Health Evaluation; AUROC: area under receiver operating characteristic curve; AUPRC: area under precision recall curve. The subplots on the left side were generated using the most abnormal values in the 24-h lookback period, whereas the plots on the right side were generated using the most recent values. The plotted values represent median and 95% confidence intervals generated through 1000 bootstrap samples.

For the discrimination of sepsis versus nonsepsis, performance of NEWS was nearly identical to that of NEWS 2, both of which were superior to all other EWS (Figure 1). As expected, performance for all EWS declines as the score predicts further ahead of index time, and continues to improve postindex time. Using the most abnormal value in the lookback period was significantly better than using the most recent value for all EWS. There was minimal difference in performance when using an alternate lookback period of 12 h (Figure 1, Supplementary Figure S1).

At index time, NEWS 2 had the highest AUROC and AUPRC (0.803 [0.795–0.812] and 0.130 [0.121–0.140], Table 2). Using the validated thresholds, NEWS 2 also had the highest recall (0.758 [0.736–0.778]) but qSOFA had the highest specificity (0.950 [0.948–0.952]), precision (0.184 [0.169–0.198]), and F1 score (harmonic mean of precision and recall, 0.236 [0.220–0.253]). The results of using alternate thresholds are shown in Supplementary Table S2.

**Table 2.** Early warning score performance at time of sepsis onset

EWS	AUROC	AUPRC	Threshold	Recall (sensitivity)	Specificity	Precision (PPV)	F1 Score
APACHE II	0.654	0.066	15	0.400	0.801	0.064	0.110
	(0.643–0.665)	(0.060–0.071)		(0.374–0.426)	(0.797–0.805)	(0.059–0.069)	(0.102–0.118)
MEWS	0.772	0.118	4	0.470	0.885	0.121	0.192
	(0.763–0.781)	(0.110–0.127)		(0.444–0.495)	(0.882–0.887)	(0.113–0.129)	(0.181–0.205)
NEWS	0.803	0.130	5	0.757	0.712	0.081	0.147
	(0.795–0.811)	(0.120–0.139)		(0.735–0.777)	(0.707–0.716)	(0.077–0.086)	(0.140–0.155)
NEWS 2	**0.803**	**0.130**	5	**0.758**	0.711	0.081	0.147
	**(0.795**–**0.812)**	**(0.121**–**0.140)**		**(0.736**–**0.778)**	(0.707–0.715)	(0.077–0.086)	(0.139–0.155)
SIRS	0.738	0.090	2	0.672	0.720	0.075	0.135
	(0.729–0.748)	(0.084–0.096)		(0.648–0.694)	(0.716–0.724)	(0.071–0.080)	(0.128–0.143)
SOFA	0.674	0.063	2	0.706	0.557	0.051	0.095
	(0.664–0.685)	(0.059–0.068)		(0.683–0.728)	(0.552–0.561)	(0.048–0.054)	(0.090–0.101)
qSOFA	0.754	0.100	2	0.330	**0.950**	**0.184**	**0.236**
	(0.745–0.763)	(0.092–0.106)		(0.308–0.355)	**(0.948**–**0.952)**	**(0.169**–**0.198)**	**(0.220**–**0.253)**

F1: harmonic mean of recall and precision; SIRS: Systemic Inflammatory Response Syndrome; qSOFA: quick Sequential Organ Failure Assessment; NEWS: National Early Warning Score; MEWS, Modified Early Warning Score; SOFA: Sequential Organ Failure Assessment; APACHE: Acute Physiology and Chronic Health Evaluation. Bolded values indicate best performance.

Values represent median and 95% confidence interval from 1000 bootstrap samples.

**Figure 2.**
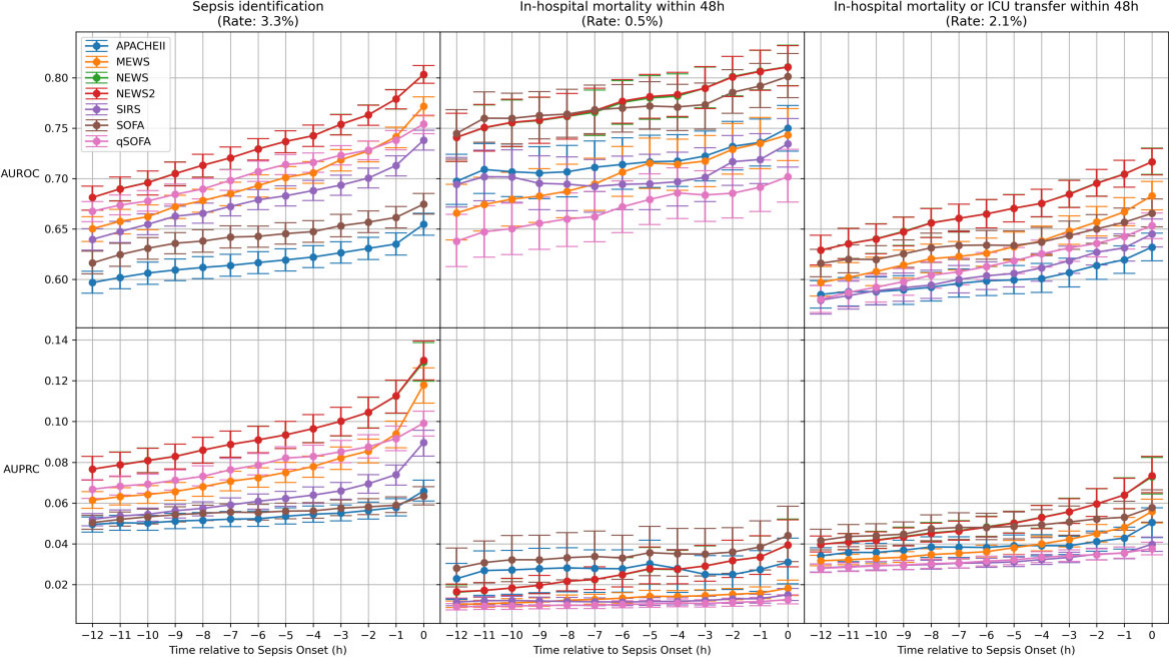
Early warning score performance for secondary outcomes. ICU: intensive care unit; SIRS: systemic inflammatory response syndrome; qSOFA: quick Sequential Organ Failure Assessment; NEWS: National Early Warning Score; MEWS: Modified Early Warning Score; SOFA: Sequential Organ Failure Assessment; APACHE: Acute Physiology and Chronic Health Evaluation; AUROC: Area Under Receiver Operating Characteristic Curve; AUPRC: Area Under Precision Recall Curve. The plots were generated using the most abnormal values in the 24-h lookback period. The plotted values represent median and 95% confidence intervals generated through 1000 bootstrap samples.

For the prediction in-hospital mortality within 48 h of index time, NEWS, NEWS 2, and SOFA had similar AUROC (∼0.81 at index time), which were superior to those of other EWS (Figure 2). For the prediction of either in-hospital mortality or ICU transfer within 48 h of index time, NEWS and NEWS 2 performed better (AUROC: ∼0.71, AUPRC: ∼0.07) compared to all other compared EWS.

## DISCUSSION

In this large retrospective analysis of EWS performance on sepsis discrimination in the general ward setting, patients who met Sepsis-3 criteria were older and had more medical comorbidities compared to other patients in the general ward. This sepsis cohort also had a higher level of acuity, length of stay, and rates of in-hospital mortality (Table 1).

Among the compared EWS and patient acuity scoring systems, NEWS 2 had the highest discriminatory capability throughout the assessed time points, including at the time of onset (Figure 1, Table 2). NEWS performed nearly identically to NEWS 2, which was followed by MEWS, qSOFA, and SIRS. Six hours prior to index time, a time when clinical action could change patient outcomes, NEWS 2 performance was ∼0.74 compared to ∼0.80 at onset. Due to the low prevalence of sepsis (3.3%), the AUPRC was <0.15 for all EWS at all time points preceding index time, is reflected in the low positive predictive value (PPV) across all EWS, which represents a propensity for high rates of false positives (Table 2). While it is possible to improve PPV through changing the threshold, it comes at the expense of reducing sensitivity (Supplementary Table S2).

The relatively poor performance of SOFA and APACHE II likely reflects the lower rate of vital sign and laboratory data collection available to patients on the hospital floor, as these tools were originally designed for the ICU setting and as patient acuity scores, not EWS. Such scores relying on infrequently measured variables (eg, arterial blood gases) appear to translate poorly to the general ward setting, as would be expected.

As seen in Figure 1, time-to-onset has a significant impact on the predictability of sepsis, and thus the performance of prediction tools. However, identification of sepsis onset time is not defined in the Sepsis-3 criteria and is prone to disagreement, which can significantly alter the results.^7^^,^^12^^,^^18^

Studies comparing EWS are heterogeneous in their experimental design, especially in identifying the time-at-risk interval from which measurements are gathered for the control population. Methods include the usage of random time intervals, full encounters, or the first 24 h of admission.^19–21^ To calculate the discriminatory ability of EWS surrounding sepsis onset, it was necessary to assign an index time for controls, and to minimize bias introduced by the duration of hospitalization, sepsis and nonsepsis cohorts were matched on time-to-index time. As a result, however, the ratio of sepsis to nonsepsis patients may not reflect the full set of hospital stays, favoring a sicker nonsepsis cohort compared to that if sampled randomly or taken whole.

While none of the compared EWS were used for the study population during the study period, a locally developed sepsis alert tool was used during the study period.^22^ Thus compared EWS that share variables with the tool may be biased towards better performance.

Surprisingly, the update from NEWS to NEWS 2 had a nearly unnoticeable impact on the performance. Many of the changes described in the report, however, address concerns not directly relating to the score calculations, but to the usage of the score.

The limitations of this study are as follows: first, this is a single-center study at a large academic medical center and its patient population and culture-of-practice may preclude widespread generalization. Second, the retrospective nature of this study may yield EWS performance metrics different from those obtained from a prospective trial. Third, the choice of sepsis definition used may have resulted in biased performance metrics of EWS, especially for qSOFA which is used in the Sepsis-3 consensus definition. Fourth, this study evaluates only sepsis that developed on the general ward within 14 days of hospitalization and does not include patients with surgery within 72 h. Further evaluation of EWS in these specific populations may provide additional insight into their utility as a sepsis surveillance tool. Fifth, mental status—a variable used in all scores except SIRS—was not available as a discrete element during the study period and was assumed normal consistent with prior reports.^7^

## CONCLUSION

In this large, retrospective, single-center study with 45 776 unique encounters, sepsis occurred in 3.3% of all hospital admissions, yielding a longer length of hospitalization and a higher rate of in-hospital mortality. EWS and patient acuity scores—APACHE II, qSOFA, MEWS, NEWS, NEWS 2, and SOFA—had low discriminative ability for sepsis, leaving room for more computationally advanced approaches.

## SUPPLEMENTARY MATERIAL

Supplementary material is available at *Journal of the American Medical Informatics Association* online.

## CONTRIBUTORS

S.C.Y., performed the data analysis, generated the figures and tables and contributed significantly to the crafting of the manuscript. N.S., contributed to the conceptualization of the project and assisted in data analysis of the EWS performance metrics. K.B., contributed to the conceptualization of this project and analyzed the clinical data. A.G., contributed to the conceptualization of the project and assisted in data analysis of the EWS performance metrics. A.M.L., analyzed and interpreted the data regarding EWS performance. M.H.K., contributed to the conceptualization of the project and assisted in data analysis of the EWS performance. P.R.O.P., played a significant role in the conceptualization of this project and analyzed and interpreted the data regarding EWS performance. A.P.M., contributed to the conceptualization of the project, assisted in clinical data and EWS performance metric review and drafted the manuscript. All authors read and approved the final manuscript.

## Conflict of interest statement

Marin H. Kollef is a consultant for Pfizer and Merck. The rest have no conflicts of interest to disclose.

## ETHICS STATEMENT

This study was approved with a waiver of consent by the Intuitional Review Board from Washington University in St. Louis prior to the commencement of this study (IRB#201804121).

## Data availability

The datasets analyzed during the current study are not publicly available due to privacy concerns.

## Supplementary Material

ooab062_Supplementary_Data
