# Supplementary material for: Comparison of early warning scores for sepsis early identification and prediction in the general ward setting
Source: JAMIA Open. 2021 Aug 2;4(3):ooab062. doi: 10.1093/jamiaopen/ooab062 (PMC8607822; doi:10.1093/jamiaopen/ooab062)
Supplement: ooab062_Supplementary_Data [file ooab062_supplementary_data.docx]

**Supplementary Online Content**

[eTable 1. Missing Data 2](#_Toc74592968)

[eTable 2. Sensitivity Analysis: Sepsis Identification at Alternate Thresholds. 3](#_Toc74592969)

[eFigure 1: Sensitivity analysis: Alternate Lookback Period (12-hour lookback) 4](#_Toc74592970)

[eAppendix I: Detailed criteria for Sepsis-3 definition 5](#_Toc74592971)

[eAppendix II: Time-to-index time sampling 6](#_Toc74592972)

[eAppendix III: Early Warning Score Calculation Implementation Details 7](#_Toc74592973)

[eAppendix IV: Data preprocessing and mapping 9](#_Toc74592974)

# eTable 1. Missing Data

| **Variable** | **Usage in EWS**^b^ | | | | | | | **Total** | | **Sepsis** | | **Non-sepsis** | |
| --- | --- | --- | --- | --- | --- | --- | --- | --- | --- | --- | --- | --- | --- |
|  | **SIRS** | **NEWS** | **NEWS 2** | **MEWS** | **SOFA** | **qSOFA** | **APACHE II** | **Measurements per encounter** | **Missing %** | **Measurements per encounter** | **Missing %** | **Measurements per encounter** | **Missing %** |
| **A-a gradient** |  |  |  |  |  |  |  | 0 (0 - 0) | 96.4 | 0 (0 - 0) | 87.6 | 0 (0 - 0) | 96.7 |
| **Bilirubin** |  |  |  |  |  |  |  | 1 (1 - 2) | 23.4 | 2 (1 - 4) | 14.1 | 1 (1 - 2) | 23.7 |
| **Creatinine** |  |  |  |  |  |  |  | 5 (3 - 7) | 0 | 8 (5 - 12) | 0 | 4 (3 - 7) | 0 |
| **FiO2** |  |  |  |  |  |  |  | 0 (0 - 13) | 58.8 | 22 (1 - 59) | 24.4 | 0 (0 - 11) | 60 |
| **HCT** |  |  |  |  |  |  |  | 5 (3 - 8) | 0 | 9 (6 - 13) | 0 | 5 (3 - 8) | 0 |
| **Heart rate** |  |  |  |  |  |  |  | 29 (16 - 58) | 0 | 82 (46 - 133) | 0 | 28 (16 - 55) | 0 |
| **MAP** |  |  |  |  |  |  |  | 26 (14 - 51) | 0 | 72 (40 - 117) | 0 | 25 (14 - 49) | 0 |
| **PCO2**^a^ |  |  |  |  |  |  |  | 0 (0 - 0) | 84 | 0 (0 - 2) | 56.4 | 0 (0 - 0) | 84.9 |
| **P-F ratio** |  |  |  |  |  |  |  | 0 (0 - 13) | 56.8 | 24 (2 - 61) | 21.3 | 0 (0 - 12) | 58 |
| **PLT** |  |  |  |  |  |  |  | 5 (3 - 8) | 0 | 9 (6 - 12) | 0 | 5 (3 - 7) | 0 |
| **PO2** |  |  |  |  |  |  |  | 0 (0 - 0) | 84 | 0 (0 - 2) | 56.4 | 0 (0 - 0) | 84.9 |
| **Potassium** |  |  |  |  |  |  |  | 5 (3 - 8) | 0.1 | 9 (6 - 13) | 0 | 5 (3 - 8) | 0.1 |
| **Respiratory rate** |  |  |  |  |  |  |  | 25 (14 - 50) | 0 | 73 (40 - 120) | 0 | 25 (14 - 48) | 0 |
| **SBP** |  |  |  |  |  |  |  | 26 (14 - 51) | 0 | 72 (40 - 117) | 0 | 25 (14 - 49) | 0 |
| **Sodium** |  |  |  |  |  |  |  | 5 (3 - 8) | 0 | 8 (5 - 13) | 0 | 5 (3 - 7) | 0 |
| **SpO2** |  |  |  |  |  |  |  | 25 (13 - 49) | 0 | 70 (39 - 119) | 0 | 24 (13 - 47) | 0 |
| **Temperature** |  |  |  |  |  |  |  | 22 (12 - 38) | 0 | 45 (29 - 68) | 0 | 21 (12 - 37) | 0 |
| **WBC** |  |  |  |  |  |  |  | 5 (3 - 8) | 0 | 9 (6 - 12) | 0 | 5 (3 - 7) | 0 |
| **pH**^a^ |  |  |  |  |  |  |  | 0 (0 - 0) | 84 | 0 (0 - 2) | 56.4 | 0 (0 - 0) | 84.9 |
| **GCS** |  |  |  |  |  |  |  | 0 (0 - 0) | 100 | 0 (0 - 0) | 100 | 0 (0 - 0) | 100 |
| **AVPU** |  |  |  |  |  |  |  | 0 (0 - 0) | 100 | 0 (0 - 0) | 100 | 0 (0 - 0) | 100 |
| Abbreviations: SIRS, Systemic Inflammatory Response Syndrome; qSOFA: quick Sequential Organ Failure Assessment; NEWS, National Early Warning Score; MEWS, Modified Early Warning Score; SOFA, Sequential Organ Failure Assessment; APACHE, Acute Physiology and Chronic Health Evaluation; A-a gradient, Arterial-alveolar gradient; FiO2, Fraction of inspired oxygen; HCT, Hematocrit; MAP, Mean Arterial Pressure; PCO2, Partial pressure of carbon dioxide; PO2, Partial pressure of oxygen; P-F ratio, PaO2 -FiO2 ratio; PLT, Platelets; SBP, Systolic Blood Pressure; SpO2, Oxygen saturation; WBC, White Blood Cell; GCS, Glasgow Coma Scale; AVPU, Alert Verbal Pain Unresponsive scale. | | | | | | | | | | | | | |
| ^a^pH and PCO2 were used to establish hypercapnic respiratory failure status for NEWS 2. | | | | | | | | | | | | | |
| ^b^The green shading indicates that the variable in the corresponding row is used in the EWS in the corresponding column. | | | | | | | | | | | | | |

# eTable 2. Sensitivity Analysis: Sepsis Identification at Alternate Thresholds.

| **EWS** | **Threshold** | **Recall (Sensitivity)** | **Specificity** | **Precision (PPV)** | **F1 Score** |
| --- | --- | --- | --- | --- | --- |
| **APACHEII** | 13 | 0.559 (0.536 - 0.585) | 0.659 (0.655 - 0.664) | 0.053 (0.049 - 0.056) | 0.096 (0.090 - 0.102) |
|  | 14 | 0.494 (0.470 - 0.520) | 0.735 (0.731 - 0.739) | 0.059 (0.055 - 0.063) | 0.106 (0.099 - 0.113) |
|  | **15** | **0.401 (0.376 - 0.427)** | **0.801 (0.797 - 0.805)** | **0.064 (0.059 - 0.069)** | **0.110 (0.102 - 0.119)** |
|  | 16 | 0.320 (0.295 - 0.346) | 0.858 (0.855 - 0.861) | 0.071 (0.064 - 0.078) | 0.116 (0.106 - 0.127) |
|  | 17 | 0.250 (0.227 - 0.272) | 0.902 (0.899 - 0.905) | 0.079 (0.072 - 0.088) | 0.120 (0.109 - 0.132) |
| **MEWS** | 2 | 0.881 (0.865 - 0.898) | 0.459 (0.455 - 0.464) | 0.052 (0.049 - 0.055) | 0.099 (0.094 - 0.104) |
|  | 3 | 0.697 (0.673 - 0.721) | 0.740 (0.736 - 0.744) | 0.083 (0.079 - 0.088) | 0.148 (0.141 - 0.157) |
|  | **4** | **0.471 (0.446 - 0.495)** | **0.885 (0.882 - 0.887)** | **0.121 (0.113 - 0.130)** | **0.193 (0.181 - 0.205)** |
|  | 5 | 0.286 (0.263 - 0.308) | 0.955 (0.953 - 0.957) | 0.176 (0.161 - 0.192) | 0.218 (0.200 - 0.235) |
|  | 6 | 0.154 (0.135 - 0.173) | 0.983 (0.982 - 0.984) | 0.234 (0.205 - 0.263) | 0.186 (0.163 - 0.207) |
| **NEWS** | 3 | 0.919 (0.904 - 0.932) | 0.440 (0.435 - 0.444) | 0.053 (0.050 - 0.055) | 0.099 (0.095 - 0.104) |
|  | 4 | 0.852 (0.834 - 0.870) | 0.590 (0.586 - 0.595) | 0.066 (0.062 - 0.069) | 0.122 (0.116 - 0.128) |
|  | **5** | **0.757 (0.734 - 0.781)** | **0.711 (0.707 - 0.715)** | **0.082 (0.077 - 0.086)** | **0.147 (0.140 - 0.155)** |
|  | 6 | 0.651 (0.625 - 0.674) | 0.800 (0.797 - 0.804) | 0.099 (0.093 - 0.105) | 0.172 (0.163 - 0.182) |
|  | 7 | 0.548 (0.521 - 0.574) | 0.867 (0.864 - 0.870) | 0.122 (0.114 - 0.131) | 0.200 (0.188 - 0.212) |
| **NEWS2** | 3 | 0.919 (0.904 - 0.932) | 0.440 (0.435 - 0.444) | 0.053 (0.050 - 0.055) | 0.099 (0.095 - 0.104) |
|  | 4 | 0.852 (0.834 - 0.870) | 0.590 (0.585 - 0.595) | 0.066 (0.062 - 0.069) | 0.122 (0.116 - 0.128) |
|  | **5** | **0.758 (0.734 - 0.781)** | **0.711 (0.707 - 0.715)** | **0.082 (0.077 - 0.086)** | **0.147 (0.139 - 0.155)** |
|  | 6 | 0.653 (0.628 - 0.677) | 0.800 (0.796 - 0.804) | 0.100 (0.093 - 0.106) | 0.173 (0.163 - 0.182) |
|  | 7 | 0.549 (0.522 - 0.576) | 0.867 (0.863 - 0.870) | 0.122 (0.114 - 0.130) | 0.200 (0.187 - 0.212) |
| **SIRS** | 1 | 0.904 (0.890 - 0.919) | 0.328 (0.323 - 0.332) | 0.044 (0.041 - 0.046) | 0.083 (0.079 - 0.087) |
|  | **2** | **0.671 (0.648 - 0.695)** | **0.720 (0.716 - 0.724)** | **0.075 (0.070 - 0.080)** | **0.135 (0.127 - 0.143)** |
|  | 3 | 0.309 (0.284 - 0.331) | 0.937 (0.934 - 0.939) | 0.141 (0.129 - 0.153) | 0.194 (0.177 - 0.209) |
| **SOFA** | 1 | 0.862 (0.844 - 0.879) | 0.344 (0.340 - 0.349) | 0.043 (0.040 - 0.045) | 0.081 (0.077 - 0.085) |
|  | **2** | **0.705 (0.682 - 0.728)** | **0.557 (0.552 - 0.561)** | **0.051 (0.048 - 0.054)** | **0.095 (0.090 - 0.101)** |
|  | 3 | 0.524 (0.500 - 0.549) | 0.730 (0.726 - 0.734) | 0.062 (0.058 - 0.066) | 0.110 (0.103 - 0.118) |
|  | 4 | 0.350 (0.327 - 0.376) | 0.843 (0.839 - 0.846) | 0.070 (0.065 - 0.076) | 0.117 (0.108 - 0.127) |
| **qSOFA** | 1 | 0.787 (0.766 - 0.808) | 0.641 (0.637 - 0.646) | 0.069 (0.065 - 0.073) | 0.127 (0.120 - 0.134) |
|  | **2** | **0.331 (0.308 - 0.356)** | **0.950 (0.948 - 0.952)** | **0.184 (0.170 - 0.199)** | **0.237 (0.220 - 0.253)** |
| Abbreviations: F1, Harmonic mean of recall and precision; SIRS, Systemic Inflammatory Response Syndrome; qSOFA: quick Sequential Organ Failure Assessment; NEWS, National Early Warning Score; MEWS, Modified Early Warning Score; SOFA, Sequential Organ Failure Assessment; APACHE, Acute Physiology and Chronic Health Evaluation. | | | | | |
| Values represent median and 95% confidence interval from 1,000 bootstrap samples. | | | | | |
| Bolded rows indicate the threshold values used for primary analysis (Table 1). | | | | | |

# eFigure 1: Sensitivity analysis: Alternate Lookback Period (12-hour lookback)


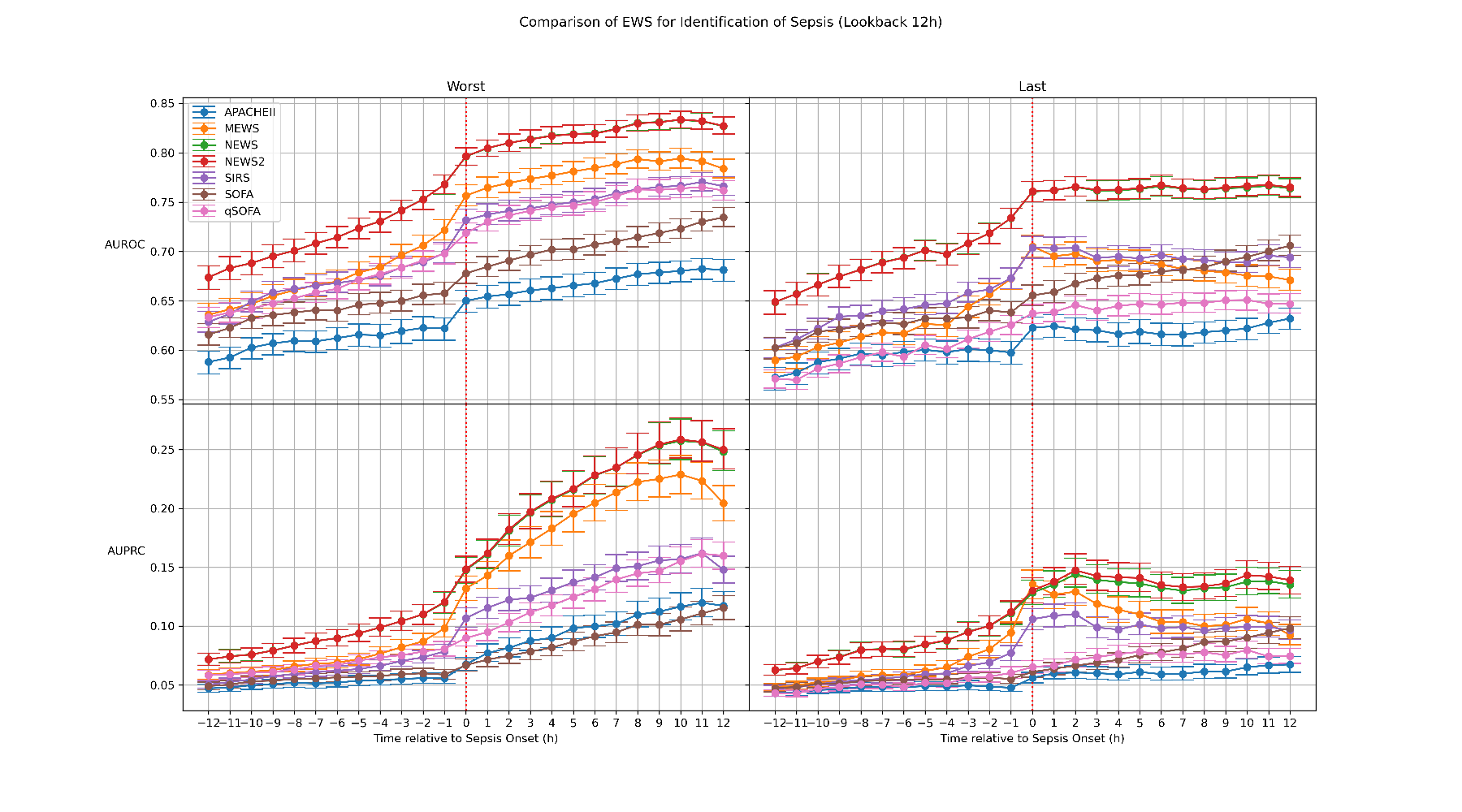


Abbreviations: SIRS, Systemic Inflammatory Response Syndrome; qSOFA: quick Sequential Organ Failure Assessment; NEWS, National Early Warning Score; MEWS, Modified Early Warning Score; SOFA, Sequential Organ Failure Assessment; APACHE, Acute Physiology and Chronic Health Evaluation; AUROC, Area Under Receiver Operating Characteristic Curve; AUPRC, Area Under Precision Recall Curve.

Instead of the 24-hour lookback period of in the primary analysis, 12-hour lookback period was used.

The subplots on the left side were generated using the most abnormal values in the lookback period, whereas the plots on the right side were generated using the most recent values.

The plotted values represent median and 95% confidence intervals generated though 1,000 bootstrap samples.

# eAppendix I: Detailed criteria for Sepsis-3 definition

1. **Suspicion of infection**: Consistent with the published Sepsis-3 criteria (Seymour, JAMA, 2016), suspicion of infection required either: 1) antibiotics within 72 hours of culture; or 2) culture within 24 hours of antibiotic administration. Time of suspected infection was set as the earlier of either occurrence.
2. **Antimicrobials**: Consistent with the source publication, all oral and IV antibiotics were included, and one-time perioperative antibiotics were excluded.
3. **Cultures**: Consistent with the source publication, all bacterial, fungal, viral and parasitic cultures as well as C. diff assays from the following sites were included: abdomen, bronchoalveolar lavage, blood, bone, cerebral spinal fluid, catheters/devices, pleural space, skin/tissue, stool, urinary tract.
4. **Response to infection:** Consistent with the Sepsis-3 publication, to qualify as a sepsis event, qSOFA ≥ 2 was required in the time window around suspicion of infection (48 hours before to 24 hours after).

# eAppendix II: Time-to-index time sampling

**
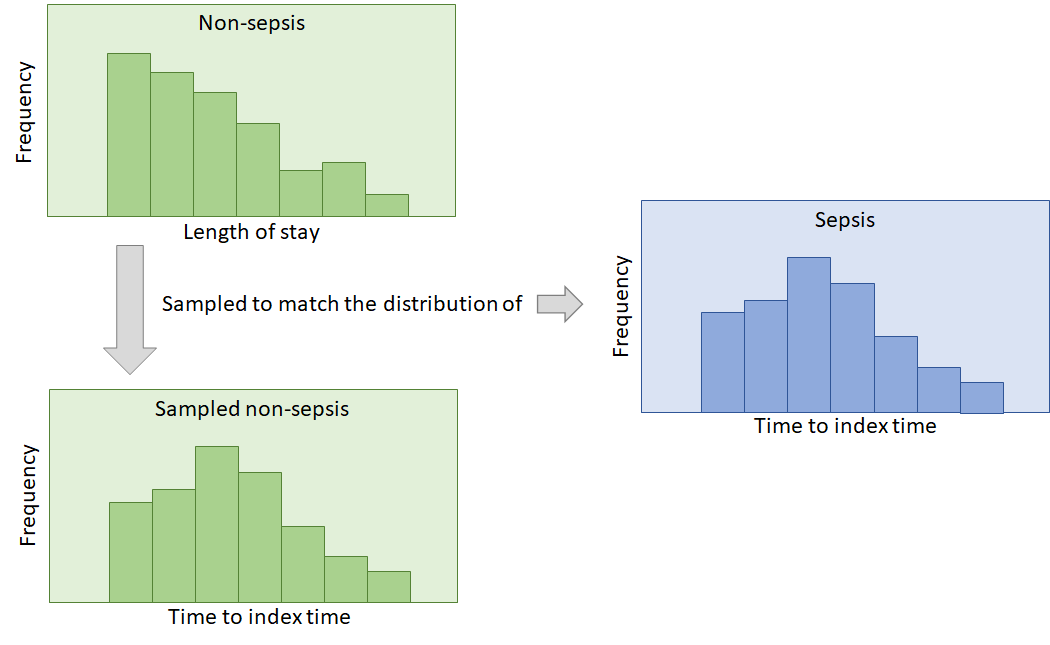
**

Based on the sepsis cohort and the associated time-to-index time, the non-sepsis cohort was subsampled and assigned an index time such that the time-to-index time distributions were similar between the two resultant cohorts. Each 3-hour time-to-index time bin in the sepsis time-to-index time histogram was used to identify a set ratio of eligible non-sepsis patients who were subsequently given a random time-to-index time between the bin boundaries. The resultant effect is the equivalent shape of histograms between the sepsis and subsampled non-sepsis cohort. The efficacy of the matching procedure is evidenced by the non-significant difference in time-to-index time between the two cohorts (Table 1).

# eAppendix III: Early Warning Score Calculation Implementation Details

1. Systemic Inflammatory Response Syndrome (SIRS) was calculated based on the original manuscript (Bone, Chest, 1992) as (1) temperature > 38°C or < 36°C; (2) heart rate > 90 bpm; (3) respiratory rate > 20 breathes per minute or PaCO2 < 32 mm Hg; and (4) white blood cell count > 12,000/ cu mm or < 4,000/cu mm.
2. NEWS was calculated based on the original report (Royal College of Physicians, 2012) using respiration rate, oxygen saturation, supplemental oxygen status, temperature, systolic blood pressure, heart rate, and level of consciousness. Because AVPU (Alert Verbal Pain Unresponsive scale) was not available (eTable 1), it was assumed normal for the purpose of score calculation.
3. NEWS 2 was calculated based on the updated report (Royal College of Physicians, 2017) which is nearly identical to NEWS except for a new oxygen saturation score scale for patients with hypercapnic respiratory failure. Hypercapnic respiratory failure status was determined by concomitant pH < 7.35 and PCO2 > 45 mm Hg.
4. MEWS was calculated based on the original manuscript (Subbe, QJM, 2001) using systolic blood pressure, heart rate, respiratory rate, temperature, and AVPU score. Because AVPU was not available, it was assumed normal for the purpose of score calculation.
5. SOFA was calculated based on the original manuscript (Vincent, Intensive Care Med, 1996) using P-F (PO2-FiO2) ratio, platelet count, bilirubin, mean arterial pressure, vasopressor administration, creatinine, and GCS (Glasgow Coma Scale). PF-ratio was imputed as described in eAppendix IV. Because GCS was not available, it was assumed normal for the purpose of score calculation.
6. qSOFA was calculated based on the original manuscript (Seymour, JAMA, 2016) using systolic blood pressure, respiratory rate, and GCS. Because GCS was not available, it was assumed normal for the purpose of score calculation.
7. APACHE II was calculated based on the original manuscript (Knaus, Crit Care Med, 1985). The “Chronic Health Points” were determined through diagnosis ICD codes, and all patients were assumed to be non-operative or emergency post-operative, not elective post-operative patients. The “Acute Physiology Score” was based on temperature, mean arterial pressure, heart rate, respiratory rate, FiO2, A-a gradient, PaO2, pH, sodium, potassium, creatinine, hematocrit, white blood cell count, and GCS. Because GCS was not available, it was assumed normal for the purpose of score calculation.

eAppendix IV: Data preprocessing and mapping

Raw clinical data were mapped to cogent clinical concepts through a combination of informatics approaches and subject matter expert manual review.

Certain data elements were not present or partially present, but were able to be derived from related data elements:

- BMI = weight (kg) / (height (m))^2^. BMI was explicitly present for 35.3% of the study population, was able to be calculated for 91.8%, and was ultimately available for 92.0%.
- FiO2 was available explicitly but was also calculated whenever there was oxygen flow documentation according to the following formula: oxygen flow x 3.5 + 21.
- PaO2 - FiO2 ratio (P-F ratio) was calculated whenever there was documentation of either PaO2 or FiO2. From each documentation, we looked back 24 hours for the latest complement documentation (PaO2 for FiO2 and vice versa) to calculate the ratio. If a complement FiO2 could not be found for PaO2, FiO2 was assumed to be 21%. If a complement PaO2 could not be found for PaO2, PaO2 was calculated using the following formula: 100 – Age (years) * 0.3
